# Relationship between relaxin‐2 levels in serum and mode of conception in twin gestations: A prospective cohort study

**DOI:** 10.1111/jog.16190

**Published:** 2024-12-23

**Authors:** Angela Silvano, Oumaima Ammar, Astrid Parenti, Noemi Strambi, Viola Seravalli, Daniele Bani, Mariarosaria Di Tommaso

**Affiliations:** ^1^ Department of Health Sciences, Division of Obstetrics and Gynecology Careggi Hospital, University of Florence Florence Italy; ^2^ Department of Health Sciences Clinical Pharmacology and Oncology Section, University of Florence Florence Italy; ^3^ Department of Clinical and Experimental Medicine Research Unit of Human Histology & Embryology, University of Florence Florence Italy

**Keywords:** assisted reproductive technologies, maternal and neonatal outcomes, relaxin, spontaneous conception, twin pregnancies

## Abstract

**Aim:**

Relaxin is a peptide hormone commonly associated with pregnancy when it is thought to play a role in modulating various physiological processes to optimize maternal–fetal adaptation. In twin pregnancies these adaptive requirements are higher than in singleton pregnancies, therefore it is important to understand how circulating relaxin behaves in such conditions. This prospective cohort study aims to determine the serum relaxin‐2 levels throughout gestation in twin pregnancies and to investigate its association with the mode of conception.

**Methods:**

Blood samples were collected during each trimester of gestation from 26 women with twin pregnancies obtained through spontaneous conception (SC, *n* = 18) or assisted reproductive technologies, specifically through intracytoplasmic sperm injection (ART, *n* = 8). Serum relaxin‐2 levels were measured by a highly sensitive ELISA method.

**Results:**

The results indicated that serum relaxin‐2 level peaks in the first trimester (491.05 ± 207.41 pg/mL), then decreases in the second trimester (446.27 ± 180.4 pg/mL; *p* < 0.057) and in the third trimester (422.19 ± 194.30 pg/mL; *p* < 0.025). Interestingly, the serum relaxin‐2 level was higher in the spontaneous conception group with respect to the assisted reproductive technologies group (*p* < 0.001), when measured at each trimester of gestation. In addition, the multivariate regression analysis showed that only the assisted reproductive technologies had a significant impact on serum levels of relaxin‐2 (*p* < 0.001), and no significant association was found with other women's clinical and demographic characteristics.

**Conclusions:**

These findings extend the current knowledge about the pattern of circulating relaxin‐2 throughout gestation in twin pregnancies, providing a sensitive measurement of serum relaxin‐2 levels and a description of its putative physiological roles in humans.

## INTRODUCTION

Relaxin (RLX) is a peptide hormone discovered in 1926 by Frederick Hisaw, who observed that the injection of serum from pregnant guinea pigs or rabbits into female guinea pigs induced relaxation of the interpubic ligament.^1^ It was named for this effect following its isolation from the corpus luteum of pregnant swine.^2^

RLX has prominent structural homologies with insulin; however, gene analysis along phylogenesis has ascertained that RLX diverged from insulin early in vertebrate evolution, as indicated by different receptors, signal transduction mechanisms, and biological functions.^3^, ^4^ On this ground, a specific RLX peptide hormone family has been coined, which in humans encompasses three RLXs, H1 to H3, and four insulin‐like peptides (INSL), 3–6.^5^ H2 RLX (RLX‐2) is the major circulating form produced mainly in the corpus luteum.^6^ It accounts for most of the known biological effects of this hormone on both the female reproductive system and non‐reproductive targets, including the renal and cardiovascular system and the connective tissue.^4^, ^7^, ^8^, ^9^ Focusing on RLX‐2 as a reproductive hormone in women, its circulating levels have been measured in the menstrual cycle and pregnancy.^10^, ^11^, ^12^ In the luteal phase, plasma RLX‐2 ranges between 50 and 160 pg/mL peaking 6–9 days after the LH peak, while the highest RLX‐2 levels, 0.9–1 ng/mL, are reached in women during the first trimester of spontaneous singleton or twin pregnancy.^13^, ^14^, ^15^

The mode of conception can affect the levels of maternal serum RLX‐2, as reported by previous studies examining the association between in vitro fertilization procedure and circulating RLX‐2 level.^14^, ^16^ These studies indicate that the level of RLX‐2 is lower in women undergoing assisted reproductive technologies (ART) conception, such as programmed frozen embryo transfer or donor egg recipient, with twin pregnancies in the absence of corpus luteum than in those with one or more corpus luteum.

The corpus luteum is the primary source of multiple factors regulating reproduction, including progesterone, estradiol, relaxin, and vasoactive and angiogenic molecules, which are also secreted into the circulation during the early stages of pregnancy.^17^, ^18^ Notably, most of the circulating RLX‐2 is produced by granulosa luteal cells, but the inner thecal cells are also a significant local source of RLX‐2.^18^ The luteal‐placental shift at the end of the first trimester leads to a decrease in RLX‐2 production due to the substantial structural regression of the corpus luteum at this gestational age,^19^ RLX‐2 is indeed synthesized in other tissues, such as the decidua and trophoblast to support pregnancy to end.^20^, ^21^ Circulating levels of RLX‐2 peak toward the end of the first trimester of pregnancy, then decrease and remain constant throughout the second and third.^17^, ^19^ In other mammals pre‐partum RLX‐2 surge has been observed, which accounts for the lengthening of the interpubic ligament and softening of the tissues of the birth canal aimed at facilitating delivery; this does not occur in humans. Another major difference concerns the ability of RLX‐2 to inhibit contractility of the myometrium, found in other mammals but negated in humans, indicating that the quiescent action of RLX on uterine motility is of negligible clinical relevance.^4^, ^22^

Indeed, the search for a specific role of RLX‐2 in human reproduction and pregnancy has given no conclusive results. RLX‐2 produces its major effects through the activation of its membrane receptor, RFXP1, promoting implantation, endometrial remodeling, placentation, and vascularization during the first trimester of pregnancy, to optimize the mother's adaptation to the increasing demand of the growing fetus^18^, ^23^, ^24^; its pleiotropism of effects suggests a possible function as a modulator of pregnancy. In this perspective, it seems conceivable that the pattern of circulatory RLX‐2 may change in multiple pregnancies because both hCG stimulation on the corpus luteum and the mothers' adaptation needs are set at a maximum level. Indeed, anecdotal data suggest that high serum RLX‐2 may occur in women with multiple gestations and ovarian stimulation, potentially associated with an elevated risk of premature births.^13^

The present study was designed to investigate the serum RLX‐2 levels throughout pregnancy in women with twin gestations and compare the concentrations between spontaneous pregnancies and those that resulted from assisted reproductive technologies. We also searched for any association between the RLX‐2 levels and various clinical parameters, to provide further insight into the putative role of RLX‐2 during twin gestation.

## MATERIALS AND METHODS

### Study design

This was a prospective, observational cohort study conducted at Careggi University Hospital in Florence, Italy, and involved women carrying a twin pregnancy who were followed at the Institution's Multiple Gestation Clinic. Twenty‐six women with twin pregnancies were enrolled. All patients gave written informed consent before inclusion. Both pregnancies obtained through spontaneous conception and assisted reproductive technologies were included. Exclusion criteria included monochorionic–monoamniotic twin pregnancy, first or second‐trimester pregnancy loss, termination of pregnancy, major fetal anomalies, and loss of follow‐up from antenatal care. Maternal characteristics and pregnancy outcomes were collected. The study was approved by the Institutional Ethical Committee (Ref. No. 10255/2017).

### Serum RLX assay

Blood samples were collected at each trimester of gestation and centrifuged at 750*g* for 10 min.^25^ Serum samples were stored at −80°C until further use. Serum RLX‐2 levels were measured using a Human Relaxin‐2 ELISA kit (Quantikine Immunoassay, Biotechne, USA), which is specific and does not cross‐react with relaxin 1 and 3. The detection range is 7.8–500 pg/mL. According to the manufacturer's instructions, optical density was measured at *λ* 450 nm wavelength using a Victor Nivo microplate reader (Perkin Elmer, USA). The serum RLX‐2 values were expressed as pg/mL by comparison with a standard curve.

### Statistical analysis

Continuous variables were presented using mean and standard deviation or median and interquartile range (IQR). The categorical variables were presented using absolute and relative frequencies. To compare the differences within the same group across each trimester of pregnancy, we used the Student's *t*‐test, paired‐*t*‐test. To evaluate the differences between groups, we used ANOVA with Bonferroni's multiple comparisons test or Mann–Whitney *U*‐test for continuous data, according to the Shapiro–Wilk test for normality, and the chi‐squared test for categorical data. A multivariate regression analysis was used to assess the association between the plasma levels of RLX‐2 and maternal characteristics. The statistical analyses were conducted using the SPSS version 28 software (SPSS Inc., Chicago, IL, USA). A statistically significant difference was accepted when the *p*‐value was <0.05. The study was sized considering RLX results as a primary endpoint by using G‐Power version 3.1.9.7 software. To have a power of 0.80 and an alpha error of 0.05, assuming a large effect size Cohen's *d* = 0.8, the number of subjects 8 + 18 resulted sufficient.

## RESULTS

Patients' demographics, clinical characteristics, and pregnancy outcomes are described in Table 1. Most women were Caucasian and nulliparous. The median maternal age was 36 years, and the median pre‐gestational BMI was 21.5 kg/m^2^. Regarding the mode of conception, 18 (69.2%) had conceived spontaneously, while eight women (30.8%) underwent in vitro *fertilization* (IVF), which involved intracytoplasmic sperm injection. All cases of ICSI were from autologous oocytes, and there was no case of frozen embryo transfer. Maternal characteristics and pregnancy outcomes were similar between SC and ART pregnancies (Table 1).

**TABLE 1:** Maternal characteristics and pregnancy and neonatal outcomes in the whole study cohort, and by mode of conception.

	All (*n* = 26)	Spontaneous conception (*n* = 18)	ART‐assisted pregnancies (ICSI; *n* = 8)	*p‐*value
Ethnicity				
Caucasian	23 (88.5%)	16 (89.0%)	7 (87.5%)	0.69
Asian	3 (11.5%)	2 (11.0%)	1 (12.0%)	
Parity				
0	21 (80.8%)	13 (72.2%)	8 (100%)	0.13
≥1	5 (19.2%)	5 (27.8%)		
Age (years)	36 (33.0–38.2)	35.5 (33.0–37.3)	37 (33.5–40)	0.29
Pre‐gestational BMI (kg/m^2^)	21.5 (20.7–22.3)	21.5 (20.9–23.1)	21.5 (19.5–22.1)	0.41
Smoking during pregnancy	1 (3.8%)	1	0	0.69
Monochorionic‐diamniotic pregnancy	5 (19.2%)	5 (27.8%)	0	
Dichorionic–diamniotic pregnancy	21 (80.8%)	13 (72.2%)	8 (100%)	0.13
Gestational age at sampling first trimester (weeks)	12.8 (11.5–13.3)	13.1 (11.9–13.4)	12.2 (10.1–12.8)	0.06
Gestational age at sampling second trimester (weeks)	22 (20.9–24.0)	22.15 (20.4–24.2)	21.60 (21.0–22.3)	0.53
Gestational age at sampling third trimester (weeks)	29.3 (28.1–31.0)	30.35 (28.2–31.3)	28.30 (28.0–29.2)	0.07
Cholestasis	5 (19.2%)	3 (16.7%)	2 (25%)	0.5
Gestational hypertension	1 (3.8%)	0	1 (12.5%)	0.31
Gestational age at delivery (weeks)	36.4 (35.1–37.2)	37 (35.5–37.3)	36.3 (36.7–36.9)	0.4
Newborns weight (g)				
First newborn	2370 (1945–2670)	2475 (2111–2720)	2070 (1840–2547)	0.18
Second newborn	2495 (2197–2665)	2495 (2335–2692)	2335 (1880–2637)	0.24
First newborn gender				
Male	10 (38.5%)	6 (33.3%)	4 (50%)	0.35
Female	16 (61.5%)	12 (66.7%)	4 (50%)	
Second newborn gender				
Male	19 (73.1%)	14 (77.8%)	5 (62.5%)	0.36
Female	7 (26.9%)	4 (22.2%)	3 (37.5%)	
1‐min Apgar index <7				
First newborn	2 (7.7%)	1 (5.6%)	1 (12.5%)	0.53
Second newborn	0 (0%)	0 (0%)	0 (0%)	
5‐min Apgar index <7				
First newborn	1 8 (3.8%)	1 (5.6%)	0 (0%)	0.69
Second newborn	0 (0%)	0 (0%)	0 (0%)	

*Note*: Categoric variables are presented as *N* (%), continuous variables as median (interquartile range). We used the Mann–Whitney *U*‐test for continuous data and the chi‐squared test for categorical data.

Abbreviations: ART, assisted reproductive technologies; BMI, body mass index; ICSI, intracytoplasmic sperm injection.

In our study cohort, there were no cases of chronic comorbidities or pre‐gestational diabetes, and no cases of fetal growth restriction, spontaneous preterm birth, or intrauterine fetal demise.

A comparison of the serum RLX‐2 levels across the three trimesters of pregnancy is reported in Table 2. The results indicated that serum relaxin‐2 level peaks in the first trimester (491.05 ± 207.41 pg/mL), then declines in the second trimester (446.27 ± 180.40 pg/mL; *p* < 0.057) and in the third trimester (422.19 ± 194.30 pg/mL; *p* < 0.025). RLX‐2 levels progressively decreased as gestation advanced.

**TABLE 2:** Serum RLX‐2 levels in the whole study cohort at each trimester of gestation.

*N* = 26	First trimester	Second trimester	Third trimester
pg/mL	491.05 ± 207.41	446.27 ± 180.40	422.19 ± 194.30
*p‐*value versus RLX‐2 first tr		0.057	0.025
*p*‐value versus RLX‐2 second tr			0.228

*Note*: Data are presented as mean ± SD. Statistical comparison: Student's paired *t*‐test.

Abbreviation: tr, trimester.

The results of the multivariate regression analysis assessing the association between plasma RLX‐2 in the first trimester and the maternal characteristics are reported in Table 3. Among the maternal characteristics considered, only the use of ART showed a significant association with plasma RLX‐2 levels in the first trimester (*p* < 0.001), and women who underwent ART had significantly (*p* < 0.001) lower RLX‐2 levels during each trimester of pregnancy compared to those with SC (Table 4).

**TABLE 3:** Multivariable regression analysis assessing the association between serum RLX‐2 in the first trimester and the maternal and pregnancy characteristics.

*N* = 26	*B*	SE	*β*	*t*	*p*	95.0% CI for *B* lower bound	95.0% CI for *B* upper bound
Predictor variables	Dependent variable: serum RLX‐2 first Tr (pg/mL)							
Age	3.71	3.13	0.07	1.18	0.25	−2.93	10.36
Pre‐gestational BMI	−9.01	7.02	−0.08	−1.28	0.22	−23.89	5.87
Ethnicity	−10.68	35.07	−0.017	−0.31	0.77	−85.03	63.67
ART (ICSI)	−435.76	27.33	−0.99	−15.94	<0.001	−493.8	−377.82
Cholestasis	−3.82	29.60	−0.007	−0.13	0.9	−66.57	58.94
Diabetes	−15.2	27.63	−0.037	−0.55	0.59	−73.77	43.37

*Note*: Values were determined by logistic regression.

Abbreviations: ART, assisted reproductive technologies; *B*, unstandardized regression coefficient; BMI, body mass index; CI, confidence interval; ICSI, intracytoplasmic sperm injection; *p*, probability value; SE, standard error for B; *t*, t‐test; *β*, standardized regression coefficient.

**TABLE 4:** Statistical comparison of the level of serum RLX‐2 between pregnancies obtained through spontaneous conception or through ART (ICSI), in each trimester of pregnancy.

Serum RLX‐2 (pg/mL)	Spontaneous conception (*N* = 18)	ART‐assisted pregnancies (ICSI; *N* = 8)	Spontaneous conception versus ART (ICSI), *p‐*value
First trimester	622.60 ± 54.90^a^	195.10 ± 41.64	<0.001
Second trimester	545.10 ± 108.00	224.00 ± 79.60	<0.001
Third trimester	517.50 ± 148.00	208.00 ± 76.40	<0.001

*Note*: Data are presented as mean ± SD. Statistical comparison between spontaneous conception and ART was performed by using ANOVA with Bonferroni's multiple comparisons test.

Abbreviations: ART, assisted reproductive technologies; ICSI, intracytoplasmic sperm injection.

^a^ Statistical comparison within the same group was performed by using Student's paired *t*‐test. Significant differences were noted in serum RLX‐2 levels between the first and second trimesters, and between the first and third trimesters (respectively, *p* = 0.0047; *p* = 0.0084) in the group of women with SC pregnancy. No significant differences were found in the group of women with ART‐assisted pregnancy.

Significant differences were found in serum RLX‐2 levels between the first and second trimesters (*p* = 0.0047), and between the first and third trimesters (*p* = 0.0084) in the group of women with SC pregnancy (Table 4). However, no significant differences throughout pregnancy were observed in the group of women undergoing ART‐pregnancy (*p* > 0.05; Table 4).

## DISCUSSION

This study shows that in twin pregnancies the serum level of RLX‐2 peaks at the first trimester and decreases progressively throughout gestation. This finding is consistent with the known trend of circulating RLX reported for human singleton pregnancies, with comparable absolute values in pg/mL.^10^, ^11^, ^12^ Our study also provides new evidence regarding the impact of the mode of conception on RLX‐2 level in pregnancy. Specifically, women who underwent ART and consequently did not develop a functional corpus luteum exhibited significantly lower or undetectable RLX‐2 levels at each trimester of pregnancy compared to those who conceived spontaneously. These findings suggest that the corpus luteum might be the primary source of circulating RLX‐2 during the first trimester of pregnancy.

Effective ART protocols require potent ovarian stimulation that inhibits the pituitary gland and blocks LH secretion.^26^ Consequently, the stimulation of post‐ovulatory granulosa cells to luteinization is impaired, resulting in a reduced secretion of progesterone and RLX. Moreover, granulosa cells, which are the main source of circulating RLX, may be inadvertently removed during oocyte retrieval, although this is typically minimized by standard techniques used.^16^, ^27^ Both these mechanisms may explain our observation of reduced RLX‐2 levels despite the presence of multiple corpora lutea. Our findings show a similar trend to those of Von Versen‐Hoynck et al., who reported that women who conceived twins through ART have significantly lower RLX‐2 and progesterone levels compared to age‐matched pregnant women who developed one or multiple corpora lutea.^16^ In contrast, other studies reported higher than normal circulating RLX.^13^ These discrepancies highlight the complexity of hormonal dynamics in ART‐conceived pregnancies.

A previous study measured serum RLX‐2 concentrations in humans singleton and twin pregnancies^14^; the researchers found that RLX‐2 levels were significantly higher in twin pregnancies compared to singleton pregnancies between 15 and 22 weeks, and they reported a mean RLX‐2 level of 0.9 ng/mL. Otherwise, there were no single pregnancies in our cohort of pregnant women, and the assay used in our research to measure RLX‐2 concentrations was different from that employed by Hanging et al. Moreover, the type of assay, the characteristics of the study population, the exact timing of blood sample collection during gestation, and the conditions of sample storage and handling can affect hormone concentrations. Standardizing these variables is essential for consistency. Furthermore, elevated circulating maternal RLX‐2 concentrations greater than 1.79 ng/mL (hyper‐relaxinemia) have been reported to be associated with premature birth (gestational age at delivery <37 weeks).^23^ Similarly, a meta‐analysis performed by Hou et al. showed that elevated maternal serum relaxin detected after 18 weeks of gestational age was associated with preterm birth in Chinese women with singleton pregnancies.^28^ The relationships between RLX‐2 and spontaneous preterm birth were also observed in twin pregnancies^29^; however, in our study, no preterm births occurred.

Previous observational studies and experimental studies, performed in vitro in isolated organs and laboratory mammals, evaluated the effect of RLX in pregnancy and suggested that this hormone could also play a supportive role in human pregnancy.^1^, ^4^, ^16^, ^30^, ^31^, ^32^ Moreover, in humans, in addition to the circulating luteal H2 RLX encoded by the *RLN2* gene, there exists an orthologous *RLN1* gene, expressed by the decidua and trophoblast, generating a paracrine H1 RLX which does not reach the bloodstream but may exert local effects, potentially promoting decidualization and facilitating embryo accommodation.^13^, ^22^, ^33^

Studies in rats and mice have suggested the potential use of RLX to improve experimentally‐induced hypertension during pregnancy.^17^, ^34^ However, the results of most clinical studies that have investigated the trend of circulating RLX throughout pregnancy, and its supplementation for the prevention of hypertensive disorder diseases have failed to confirm its protective effect.^35^, ^36^ These clinical studies could not confirm the working hypothesis of a protective role of relaxin suggested by the animal investigations, primarily because of an inadequate number of women recruited which led to weak statistics. This implies a lack of consensus or supporting data regarding RLX's function in maintaining human pregnancy. Furthermore, a previous study reported that RLX levels in the third trimester and at the time of labor were higher in twin pregnancies compared to singleton pregnancies.^37^ Future research is required to validate these findings, employing assays with higher sensitivity and specificity, such as the ELISA for the human peptide, as used in our study, instead of the radioimmunoassay test for the porcine peptide used in the above pioneer clinical studies. We recognize that a larger sample size is desirable for enhancing the statistical power and generalizability of the findings. The limitations of our study were the small sample size and the absence of a control group with singleton pregnancies.

In conclusion, pregnancy requires a finely tuned hormonal regulation to ensure optimal fetal development and well‐being of the mother.^38^, ^39^, ^40^

Relaxin‐2 plays a critical role in pregnancy by facilitating cardiovascular adaptations, promoting uterine and pelvic flexibility, and supporting fetal growth. Clinically, monitoring relaxin‐2 levels may help in identifying risks associated with preterm labor or complications from insufficient cervical remodeling. Understanding relaxin‐2's function could also inform potential therapeutic approaches, especially for managing vascular health and pregnancy complications, such as preeclampsia.^41^

In this complex mechanism, the exact function of RLX remains ill‐defined. Although its major effects on collagen remodeling, blood vessel dilation and neoangiogenesis, and inflammatory/immune cell modulation might contribute to optimizing the maternal–fetal interaction, circulating RLX‐2 does not appear to be a mandatory requirement for a successful pregnancy and normal fetal growth.^23^, ^24^ The main points of strength of the present study are the prospective design and the use of a standardized protocol for the collection and processing of samples in a single specialized laboratory, which limits the risk of technical artifacts. Its main limitation is that our cohort was too small to determine whether the lower RLX‐2 levels observed in ART pregnancies could influence pregnancy outcomes compared to spontaneously conceived pregnancies. Future studies involving larger cohorts of women from different ethnic backgrounds, conceiving spontaneously or through ART, and bearing singlet or twin fetuses, could hopefully provide a better understanding of the possible RLX's role in human pregnancy.

## AUTHOR CONTRIBUTIONS

**Angela Silvano:** Conceptualization; data curation; formal analysis; investigation; methodology; writing – original draft; writing – review and editing. **Oumaima Ammar:** Formal analysis; writing – original draft; writing – review and editing. **Astrid Parenti:** Visualization; writing – review and editing. **Noemi Strambi:** Formal analysis; writing – review and editing. **Viola Seravalli:** Validation; visualization; writing – review and editing. **Daniele Bani:** Conceptualization; validation; visualization; writing – review and editing. **Mariarosaria Di Tommaso:** Conceptualization; funding acquisition; validation; visualization; writing – review and editing.

## FUNDING INFORMATION

This study was supported by Fondazione CRF Firenze, grant number B15F21003630007, issued to Professor Mariarosaria Di Tommaso.

## CONFLICT OF INTEREST STATEMENT

The authors declare that they have no conflicts of interest.

## ETHICS STATEMENT

This study was approved by the Ethical Committee of Azienda Ospedaliero‐Universitaria Careggi (Ref. No. 10255/2017). This study was conducted in accordance with the Declaration of Helsinki, and the rights and privacy of all subjects were protected.

## CONSENT TO PARTICIPATE

Informed consent was obtained from the patient.

## Data Availability

Data available on request from the authors. The data that support the findings of this study are available from the corresponding author upon reasonable request, in accordance with the principles of privacy protection outlined in the European General Data Protection Regulation (GDPR, Regulation (EU) 2016/679).
